# Trends in population blood pressure and prevalence, awareness, treatment and control of hypertension among older persons: The 2006 & 2015 National Health and Morbidity Survey in Malaysia

**DOI:** 10.1371/journal.pone.0238780

**Published:** 2020-09-10

**Authors:** Bee Kiau Ho, Mohd Azahadi Omar, Rajini Sooryanarayana, Sazlina Shariff Ghazali, Sheleaswani bte Inche Zainal Abidin, Ambigga Krishnapillai, Suthahar Ariaratnam, Noorlaili Mohd Tohit, Nur Liana bt Abdul Majid, Muhammad Fadhli bin Mohd Yusof

**Affiliations:** 1 Bandar Botanic Health Center, Bandar Botanic, Klang, Selangor, Malaysia; 2 Sector for Biostatistic & Data Repository, National Institutes of Health, Ministry of Health Malaysia, Shah Alam, Selangor, Malaysia; 3 Family Health Development Division, Ministry of Health, Putrajaya, Malaysia; 4 Department of Family Medicine, Faculty of Medicine and Health Sciences, Universiti Putra Malaysia, Seri Kembangan, Selangor, Malaysia; 5 Malaysian Research Institute on Ageing (MyAgeing^TM^), Universiti Putra Malaysia, Seri Kembangan, Selangor, Malaysia; 6 Department of Family Medicine, Faculty of Medicine and Defence Health, National Defence University of Malaysia, Kuala Lumpur, Malaysia; 7 Department of Psychiatry, Faculty of Medicine, Universiti Technologi MARA (UiTM), Selayang Campus, Batu Caves, Selangor, Malaysia; 8 Department of Family Medicine, Faculty of Medicine, Universiti Kebangsaan Malaysia, Kuala Lumpur, Malaysia; International University of Health and Welfare, School of Medicine, JAPAN

## Abstract

**Background:**

Hypertension is one of the most important risk factors for cardiovascular diseases. Thus, it is an important public health challenge worldwide. In Malaysia, only a few studies have focused on the trends of hypertension specifically for the aging population. In view of the rapid growth of the elderly population in Malaysia, there is an urgent need to explore the condition of hypertension in this aging population. Hence, the objectives of this study were to determine the trends of population mean systolic and diastolic blood pressure (BP) levels, the prevalence, awareness, treatment and control of hypertension, and its’ associated factors among older persons in two cross-sectional national surveys that were conducted in 2006 and 2015 in Malaysia.

**Methods:**

This was a subanalysis of secondary data collected from the two cross-sectional national population-based surveys conducted in Malaysia in 2006 and 2015. Adults aged 60 and older who had participated in these two surveys were included in the study.

**Results:**

A total of 4954 (2295 males and 2659 females) and 3790 (1771 males and 2019 females) respondents completed the hypertension module surveys in 2006 and 2015, respectively. The mean age of the respondents was 68.5±6.9 years in 2006 and 68.6±7.1 years in 2015 and the difference was not significant. The prevalence of hypertension significantly reduced from 73.8% in 2006 to 69.2% in 2015 (p<0.001). Among the respondents with hypertension, the awareness, treatment and control of hypertension significantly increased from 49.7% to 60.2%, 86.7% to 91.5% and 23.3% to 44.8%, respectively, from 2006 to 2015. Logistic regression analysis showed that female sex and unemployed/retiree were significantly associated with higher hypertension prevalence in both 2006 and 2015. Being unemployed/ retiree was significantly associated with higher awareness of hypertension in both 2006 and 2015. In both 2006 and 2015, Chinese ethnicity were significantly associated with higher awareness and control of hypertension.

**Conclusions:**

The mean population BP levels and hypertension prevalence among the elderly population in Malaysia have reduced significantly over the past decade. Although the awareness, treatment and control of hypertension among older adults have improved significantly, the awareness and control rates remain suboptimal. As population aging is inevitable, appropriate public health programs and optimal treatment strategies targeting this vulnerable group are urgently needed to improve the overall awareness and control of hypertension and to prevent hypertension-related complications.

## Introduction

Hypertension is an important public health challenge as it is the leading preventable cause of premature death worldwide [1]. In 2010, 31.1% of the world’s adults had hypertension; 28.5% in high-income countries and 31.5% in low- and middle-income countries [2]. In high-income countries, the overall prevalence of hypertension has reduced and control of blood pressure (BP) has improved, likely due to the improvement of awareness, management and control of cardiovascular diseases (CVDs) risk factors [2–5]. However, in most low- and middle-income countries, there is an increasing trend of hypertension prevalence with low awareness, treatment and control. The increasing prevalence of hypertension with poor BP control has contributed to the rising epidemic of CVDs in these countries [6–9].

Hypertension is one of the most important risk factors for CVDs [10]. According to the World Health Organisation (WHO), CVDs are the number one cause of death globally and an estimated 17.7 million people died from CVDs in 2015, representing 31% of all global mortality. Over three quarters of CVD deaths take place in low and middle-income countries [11]. A previous study has shown that lowering the high BP may reduce the risk for morbidity and mortality due to CVD [12]. Therefore, effective strategies for preventing CVD requires increased prevention of hypertension, early diagnosis and timely treatment of hypertension [13].

Malaysia is experiencing rapid demographic and epidemiologic transitions. The overall prevalence of non-communicable diseases (NCDs) such as diabetes mellitus and hypertension have increased over the past few decades [14–16]. Hence, CVD has been the leading cause of both morbidity and mortality in Malaysia, responsible for 20.1% of all annual deaths in 2016 [17]. The aging population is rapidly increasing in Malaysia, older persons aged ≥65 years will constitute 7.2% of the total population by 2020 [18]. A previous study has shown that the prevalence of hypertension peaked at 74.1% among population aged 65 to 69 years in the 2011 National Health and Morbidity Survey (NHMS) in Malaysia. However, this study aimed to determine trends in prevalence, awareness and control of hypertension mainly among population ≥18 years in Malaysia [19]. Only a single study has focused on the trends of hypertension specifically for the aging population in Malaysia [20]. In view of the rapid growth of the elderly population in Malaysia, there is an urgent need to explore the prevalence of hypertension in this aging population. The objectives of this study were to determine the trend of population mean systolic BP (SBP) and diastolic BP (DBP) levels, the prevalence, awareness, treatment and control of hypertension and its’ associated factors among the older persons aged ≥60 years in two cross-sectional national surveys that were conducted in 2006 and 2015 in Malaysia.

## Methods

### Study design

National Health and Morbidity Surveys (NHMS) 2006 and 2015 were conducted by the Institute for Public Health, National Institutes of Health and funded by the Ministry of Health Malaysia. These studies were reviewed and approved by the Medical Research & Ethics Committee, Ministry of Health Malaysia. Detailed description of the sampling methods are described in the previous NHMS technical reports [14, 16]. Briefly, the study design was cross-sectional for a national population based survey with two-stage stratified random sampling throughout Malaysia. Random selection of the primary and secondary sampling units was done from a sampling frame provided by the Department of Statistics Malaysia. The primary sampling unit was the Enumeration Block (EB), within which the Living Quarters (LQ) were selected as the secondary sampling unit. All households and eligible persons within each selected LQ were included in the study. For the hypertension data, eligible respondents were 18 years and above. The estimated sample size at the national level was based on the stratification of respondents by states and by urban/ rural strata. This study was a subanalysis of secondary data collected from the two cross-sectional national population-based surveys [14, 16] conducted in 2006 and 2015 in Malaysia. The samples from 2006 and 2015 survey did not consist of the same respondents. Adults aged 60 and older who had participated in these two surveys were included in the study and a total of 8,744 older persons were included in the analysis, i.e. 4954 participants from 2006 survey and 3790 participants from 2015 study.

### Data collection and measurement

Data was collected by face to face interview with a standardized questionnaire on demographic characteristics such as age, sex, ethnicity, education, strata, marital status, occupation and household income, after obtaining written informed consent from each respondent. All household members age 18 years and above were examined for their BP. Trained nurses measured each participants’s BP according to the standardized protocol as below [21]:

- The participants were advised to refrain from smoking, eating, consuming caffeine, or engaging in physical exercise, for at least 15 minutes prior to measurement
- Seated position, back supported, arm supported
- Seated with legs uncrossed, not talking and relaxed
- The correct cuff bladder placed at heart level with the correct cuff size

Two readings of systolic and diastolic BP within 15 minutes apart were taken using Omron Digital Automatic Blood Pressure Monitor Model HEM-907 which had been validated and calibrated [22]. Blood pressure was recorded as the average reading from these two measurements.

Blood pressure results were obtained and immediately recorded in the questionnaire. Respondents were informed of the results and, if found to be hypertensive, they were referred to the nearest government health facility for further evaluation and management.

### Definitions and measurement

Hypertension was defined as SBP ≥140mmHg and/or DBP ≥90mmHg and/or previously being told to have hypertension by medical personnel. This protocol is in accordance with the Seventh Annual Report of the Joint National Committee [23].

Awareness of hypertension was defined as having ever been told to have hypertension by a medical doctor or paramedic among all hypertensive respondents.

Current treatment for hypertension was defined as respondents who were taking antihypertensive medication within two weeks of the time of interview among those who were aware of their hypertension status.

Control of hypertension was defined as having a desirable BP level (i.e. an average SBP<140 mmHg and an average DBP <90mmHg) among those who were on pharmacological treatment of hypertension. [23]

Older persons were defined as those who were 60 years and older. We further stratified them into three subgroups i.e. the young-old (ages 60–69), the middle-old (ages 70–79) and the old-old (over age 80).

### Statistical analysis

The data from the NHMS 2006 and 2015 were analysed using the Complex Samples Procedures of SPSS 21.0 for Windows. All statistical tests were two-tailed and a p value of < 0.05 was considered to be statistically significant. The sociodemographic and clinical characteristics were examined using descriptive statistics, t-tests and chi-square tests were used to examine differences in the continuous and categorical variables, respectively. The overall prevalence, awareness, treatment, and control rates in the two surveys from 2006 and 2015 were compared using a chi-square test. A logistic regression analysis was used to calculate the factors associated with the prevalence, awareness, treatment and control of hypertension in the 2006 and 2015 surveys. A comparison of findings is possible as NHMS 2006 and 2015 used similar methodology.

## Results

### General characteristics of the study population

As shown in Table 1, a total of 4954 (2295 males and 2659 females) and 3790 (1771 males and 2019 females) respondents completed the hypertension module surveys in 2006 and 2015, respectively. The mean age of the respondents was 68.5±6.9 years in 2006 and 68.6±7.1 years in 2015 and the difference was not significant. SBP and DBP has reduced significantly from 151.9±25.6 mmHg to 138.0±36.0 mmHg and 82.4±13.9 mmHg to 76.2±20.3 mmHg, respectively, from 2006 to 2015. The respondents in 2015 have significantly higher education level, household income and employment rate as compared to 2006.

**Table 1:** Sociodemographic and clinical characteristics of respondents for hypertension module among older persons aged 60 years and above in NHMS 2006 & 2015 (n = 8744).

Study variables	2006 (n = 4954)	2015 (n = 3790)	p value
	Mean ± SD	Mean ± SD	
**Age (year)**	68.5±6.9	68.6±7.1	0.740
**SBP (mmHg)**	151.9±25.6	138.0±36.0	<0.001
**DBP (mmHg)**	82.4±13.9	76.2±20.3	<0.001
	**Number (%)**	**Number (%)**	
**Age groups (y)**			
60–69	3125 (63.1)	2356 (62.2)	0.445
70–79	1418 (28.6)	1131 (29.8)	
≥ 80	411 (8.3)	303 (8.0)	
**Sex**			
Male	2295 (46.3)	1771 (46.7)	0.709
Female	2659 (53.7)	2019 (53.3)	
**Ethnicity**			
Malays	2611 (52.7)	2428 (64.1)	<0.001
Chinese	1409 (28.4)	815 (21.5)	
Indians	317 (6.4)	232 (6.1)	
Other Bumiputras	527 (10.6)	259 (6.8)	
Others	90 (1.8)	56 (1.5)	
**Education**			
Primary or below	4237 (86.7)	2726 (72.7)	<0.001
Secondary or above	651 (13.3)	1025 (27.3)	
**Strata**			
Urban	2513 (50.7)	1848 (48.8)	0.068
Rural	2441 (49.3)	1942 (51.2)	
**Marital status**			
Single	90 (1.8)	87 (2.3)	0.236
Married	3369 (68.5)	2557 (67.5)	
Widow/ Widower /divorcee	1457 (29.6)	1146 (30.2)	
**Occupation**			
Employed	1808 (37.6)	873 (43.9)	<0.001
Unemployed/ retiree	2997 (62.4)	1114 (56.1)	
**Household income (RM)**			
< 1,000	2490 (54.2)	1345 (35.5)	<0.001
1000–1999	998 (21.7)	822 (21.7)	
≥ 2000	1110 (24.1)	1623 (42.8)	

### Changes in prevalence, awareness, treatment and control of hypertension

Table 2 shows that the prevalence of hypertension significantly reduced from 73.8% to 69.2% (p<0.001) from 2006 to 2015.

**Table 2:** Prevalence, awareness, treatment and control rate of hypertension among older persons aged 60 years and above in NHMS 2006 & 2015.

Variables	2006 (n = 4954)	2015 (n = 3790)	p value
	% (CI)	% (CI)	
**Prevalence**	73.8 (72.5–75.1)	69.2 (67.1–71.2)	<0.001
**Awareness among HPT**	49.7 (48.1–51.4)	60.2 (57.5–62.8)	<0.001
**Treatment among aware**	86.7 (85.0–88.2)	91.5 (89.2–93.4)	0.001
**Control Among Treated**	23.3 (21.1–25.6)	44.8 (41.0–48.6)	<0.001

CI, confidence interval.

Among the respondents who had hypertension, 49.7% were aware of their condition in 2006, this rate significantly increased to 60.2% in 2015 (p<0.001).

From 2006 to 2015, there was a significant increase in hypertensive patients receiving treatment i.e. from 86.7% in 2006 to 91.5% in 2015 (p = 0.001).

In 2006, only 23.3% of patients with hypertension had their BP controlled, but this proportion approximately doubled to 44.8% in 2015 (p<0.001).

### Factors associated with prevalence of hypertension

Table 3 shows the multiple logistic regression analysis results of factors associated with hypertension prevalence in 2006 and 2015. We found that female sex and being unemployed/ retiree were all significantly associated with hypertension prevalence in both 2006 and 2015. In 2015, middle-old (70–79 years old) was significantly associated with hypertension prevalence.

**Table 3:** Factors associated with prevalence of hypertension among older persons aged 60 and above in NHMS 2006 & 2015.

Variables	2006 (n = 4954)	2015 (n = 3790)
	AOR (95% CI)	AOR (95% CI)
**Age groups (y)**		
60–69	1.00	1.00
70–79	1.13 (0.96–1.33)	1.50 (1.17–1.92)***
≥ 80	1.03 (0.78–1.35)	1.13(0.58–2.18)
**Sex**		
Male	1.00	1.00
Female	1.21 (1.02–1.44)*	1.33 (1.09–1.64)**
**Ethnicity**		
Malays	1.00	1.00
Chinese	0.81 (0.68–0.97)*	0.84 (0.67–1.06)
Indians	0.78 (0.58–1.04)	1.59 (1.01–2.49)*
Other Bumiputras	0.59 (0.48–0.73)***	0.92 (0.62–1.38)
Others	0.77 (0.46–1.30)	0.40 (0.18–0.93)*
**Education**		
Primary or less	1.00	1.00
Secondary or more	0.93 (0.75–1.14)	0.89 (0.73–1.09)
**Strata**		
Urban	1.00	1.00
Rural	1.14 (0.98–1.33)	0.98 (0.79–1.22)
**Marital status**		
Single	1.00	1.00
Married	0.60 (0.34–1.06)	1.53 (0.80–2.93)
Widow/ Widower /divorcee	0.76 (0.42–1.35)	1.62 (0.82–3.21)
**Occupation**		
Employed	1.00	1.00
Unemployed/ retiree	1.28 (1.09–1.52)**	1.37 (1.12–1.68)**
**Household income (RM)**		
< 1,000	1.00	1.00
1000–1999	0.95 (0.79–1.13)	0.96 (0.73–1.26)
≥ 2000	0.96 (0.80–1.14)	0.89 (0.70–1.13)

AOR, adjusted odds ratio; 95% CI, 95% confidence interval.

*p<0.05

**p<0.01

***p<0.001

### Factors associated with awareness, treatment and control of hypertension

Results of logistic regression analysis of factors associated with hypertension awareness, treatment and control in 2006 and 2015 are shown in Table 4.

**Table 4:** Factors associated with awareness, treatment and control of hypertension among older persons aged 60 years and above in NHMS 2006 & 2015.

Variables	Awareness among HPT		Treatment among aware		Control among treated	
	2006 (n = 3271)	2015 (n = 1303)	2006 (n = 1635)	2015 (n = 742)	2006 (n = 1401)	2015 (n = 683)
	AOR (95% CI)	AOR (95% CI)	AOR (95% CI)	AOR (95% CI)	AOR (95% CI)	AOR (95% CI)
**Age groups (y)**						
60–69	1.00	1.00	1.00	1.00	1.00	1.00
70–79	0.89 (0.76–1.04)	1.24 (0.94–1.63)	0.83 (0.60–1.14)	1.22 (0.60–2.49)	0.85 (0.64–1.14)	1.63 (1.14–2.32)**
≥ 80	0.44 (0.34–0.59)***	0.52 (0.24–1.12)	0.50 (0.29–0.85)*	0.66 (0.08–5.51)	1.05 (0.59–1.85)	0.66 (0.19–2.24)
**Sex**						
Male	1.00	1.00	1.00	1.00	1.00	1.00
Female	1.23 (1.03–1.46)*	0.99 (0.76–1.28)	1.21 (0.90–1.63)	0.65 (0.36–1.18)	0.76 (0.59–0.98)*	0.91 (0.64–1.30)
**Ethnicity**						
Malays	1.00	1.00	1.00	1.00	1.00	1.00
Chinese	1.23 (1.03–1.47)*	1.64 (1.23–2.19)***	2.96 (1.98–4.42)***	1.75 (0.85–3.61)	1.74 (1.32–2.31)***	1.75 (1.21–2.53)**
Indians	1.34 (0.98–1.82)	1.13 (0.73–1.77)	2.35 (1.16–4.77)*	2.18 (0.51–9.35)	2.03 (1.30–3.17)**	1.82 (1.00–3.33)
Other Bumiputras	1.14 (0.89–1.45)	2.38 (1.41–4.02)	1.15 (0.73–1.84)	0.87 (0.34–2.19)	1.52 (0.98–2.34)	1.23 (0.66–2.27)
Others	0.63 (0.36–1.09)	0.40 (0.10–1.59)	1.30 (0.38–4.51)	0.24 (0.02–2.71)	0.58 (0.13–2.54)	1.72 (0.11–27.72)
**Education**						
Primary or less	1.00	1.00	1.00	1.00	1.00	1.00
Secondary or more	1.39 (1.11–1.76)**	1.14 (0.90–1.44)	2.64 (1.40–4.98)**	1.23 (0.66–2.30)	0.87 (0.60–1.26)	1.15 (0.84–1.59)
**Strata**						
Urban	1.00	1.00	1.00	1.00	1.00	1.00
Rural	0.82 (0.70–0.97)*	0.95 (0.74–1.23)	0.86 (0.62–1.19)	1.38 (0.76–2.48)	0.84 (0.63–1.13)	1.04 (0.73–1.48)
**Marital status**						
Single	1.00	1.00	1.00	1.00	1.00	1.00
Married	1.45 (0.87–2.40)	1.77 (0.74–4.24)	1.41 (0.47–4.22)	1.14 (0.13–9.92)	0.62 (0.26–1.47)	0.48 (1.12–1.98)
Widow/ Widower /divorcee	1.33 (0.80–2.23)	1.88 (0.76–4.67)	1.29 (0.42–3.95)	1.53 (0.17–14.16)	0.68 (0.28–1.65)	0.38 (0.09–1.63)
**Occupation**						
Employed	1.00	1.00	1.00	1.00	1.00	1.00
Unemployed/ retiree	1.61 (1.34–1.93)***	1.90 (1.51–2.39)***	1.11 (0.74–1.66)	2.12 (1.24–3.62)**	0.95 (0.68–1.33)	1.07 (0.76–1.50)
**Household income (RM)**						
< 1,000	1.00	1.00	1.00	1.00	1.00	1.00
1000–1999	0.93 (0.78–1.12)	1.25 (0.91–1.72)	1.15 (0.78–1.70)	0.75 (0.37–1.54)	1.22 (0.88–1.68)	1.10 (0.71–1.71)
≥ 2000	1.06 (0.88–1.27)	1.04 (0.79–1.37)	1.21 (0.81–1.80)	1.24 (0.63–2.46)	1.14 (0.83–1.55)	1.09 (0.74–1.60)

AOR, adjusted odds ratio; 95% CI, 95% confidence interval.

*p<0.05

**p<0.01

***p<0.001

In both 2006 and 2015, being Chinese and unemployed/ retiree were significantly associated with higher awareness of hypertension. In 2006, female sex and higher education level were significantly associated with higher hypertension awareness. Older age (≥ 80 years old) and rural residents were significantly associated with lower awareness of hypertension.

We found that being Chinese or Indian and higher education were significantly associated with higher hypertension treatment in 2006. Older age (≥ 80 years old) was associated with lower treatment of hypertension. In 2015, only unemployed/retiree was significantly associated with higher hypertension treatment.

In 2006, being Chinese or Indian were significantly associated with better hypertension control. Female sex was significantly associated with poorer hypertension control in 2006. In 2015, middle-old (70–79 years old) and being Chinese were significantly associated with better hypertension control.

## Discussion

This study describes the trends in BP levels among older persons in Malaysia. The results showed that average BP levels in this population has reduced significantly over the last 10 years. From 2006 to 2015, mean SBP reduced by 13.9 mmHg and mean DBP reduced by 6.2 mmHg. The downward trends in mean SBP and DBP found in this study are consistent with the results of another recent study in the United States (US) [24]. The benefits of population BP reduction have been reported by the Framingham Heart Study investigators who found that a small reduction of 2-mm Hg in DBP in the population was associated with an estimated 17% decrease in the prevalence of hypertension, a 6% reduction in the risk of coronary heart disease and a 15% reduction in the risk of stroke [25].

This study reports the trends of hypertension prevalence, awareness, treatment and control in the past decade among older persons in Malaysia. We analysed the data on elderly participants aged ≥60 years from the two national cross-sectional surveys conducted by using the same methods in 2006 and 2015. The results showed that the prevalence of hypertension decreased, while the awareness, treatment and control of hypertension all increased significantly from 2006 to 2015.

The prevalence of hypertension among older persons in this study was 69.2% in 2015 which is similar to those reported by Guo et al. from US [3], who found that the prevalence of hypertension was 66.7% among US adults ≥ 60 years in 2009 and 2010. The downward trends in prevalence of hypertension among the older persons (from 73.8% in 2006 to 69.2% in 2015) found in this study are inconsistent with the previous study which revealed that the prevalence of hypertension remained stable from 1999 to 2010 in US. [3] Possible reasons for the downward trend of hypertension prevalence observed in this study may be due to the fact that the respondents in 2015 have higher education level, household income and employment rate as compared to 2006. Similarly, a large population-based study of a multi-ethnic Asian population conducted in Singapore between 2004 and 2010 revealed that lower educational level and being homemaker or retired/unemployed were factors significantly associated with hypertension prevalence across all ethnic groups. [26] We should enhance the effective lifestyle interventions such as healthy diet, regular exercise, alcohol and salt restriction to further reduce the hypertension prevalence among older persons in Malaysia [27].

In this study, we found that females had a higher prevalence of hypertension in 2006 and 2015, demonstrating similar trends to that reported by Wu et al. from China [28]. These results showed that older females might have higher chance of developing hypertension as compared to their counterparts. This study reports that the older persons who were unemployed or retired had a higher prevalence of hypertension in 2006 and 2015. Further analysis revealed that the unemployed/ retiree respondents were significantly older than those employed and thus it might partly explain why they had higher prevalence of hypertension. Our finding is consistent with report by Behncke et al. [29] who found that retirement significantly increased the risk of having hypertension in the English Longitudinal Study of Ageing (ELSA). Whether employment status really influences the prevalence of hypertension among older persons might be an interesting subject to be explored further in future studies.

Among the elderly participants with hypertension, the awareness, treatment and control of hypertension all significantly increased from 49.7% to 60.2%, 86.7% to 91.5% and 23.3% to 44.8% respectively, from 2006 to 2015. The rates found in the present study are higher than those of Xi et al. from China [30], who reported an increase of 38.7% to 54.3% for awareness, 30.5% to 49.0% for treatment and 6.1% to 12.0% for control from 2000 to 2009 among participants aged ≥ 60 years. However, our rates remained lower as compared to the US elderly population i.e. 73.4% to 84.0% for awareness and 34.1% to 54.9% for control from 2001 to 2010 [3]. Many factors might contribute to suboptimal awareness and control rates of hypertension. Lack of routine BP measurement was associated with low awareness of hypertension [31], poor medication adherence, and failure of health care providers to initiate or intensify treatment which were some of the barriers to BP control [32]. Given the rapid demographic transition occurring in Malaysia, public health strategies and education programs should be enhanced to meet the need of the aging population, particularly by creating higher awareness and providing more effective treatment to facilitate the optimal control of hypertension among elderly hypertensive population.

We found that older female had higher hypertension awareness but lower hypertension control. Such finding is consistent with those reported by Rahman et al. [33] from Bangladesh, who found that hypertension awareness was higher among women than men but did not translate into better antihypertensive medication practice due to gender disadvantage and inequity, thus leading to poor blood pressure control among women. Further studies need to be conducted locally to explore the possible reasons to explain the sex differences observed in this study.

We also found that older Chinese respondents had higher hypertension awareness, treatment and control as compared to their counterparts. These findings are in accordance with a recent study conducted in Singapore [34], which reported that Malay ethnicity were more likely to be unaware of being hypertensive as compared to Chinese ethnicity. Possible explanations should be explored further in future studies.

A recent study in China [28] reported that higher education level was significantly associated with hypertension awareness and treatment which is consistent with the findings of the present study. A recent multinational study [35] also reported that higher education was associated with greater awareness, treatment, and control of hypertension among the participants. Moreover, in low-income countries, hypertension awareness, treatment and control were lower in participants with lower education, most likely due to low socioeconomic status. It may lead to poorer access to care, lack of knowledge and awareness of the consequence of uncontrolled hypertension. Similarly, a recent study from China found that the higher educated respondents were more likely to have adequate hypertension related knowledge and behavior to enable them to access, evaluate, utilize and obtain important health knowledge [36]. Well-educated respondents were more likely to adopt health behavior based changes easily and have better resources to improve health behavior including a better social support.

This study found that being unemployed or retired is significantly associated with greater hypertension awareness and treatment. This is consistent with a previous study from China [36], which found that unemployed and retired respondents had higher odds of hypertension related behavior than employed workers. Employment is always thought to have a positive impact on health but unemployment is not always harmful. Retirement and unemployment can reduce occupational related stress and increase leisure time to practice health promoting activities at the same time.

Our study has some potential limitations. Firstly, as it was a cross-sectional survey, we could only observe the hypertension prevalence, awareness, treatment and control in the elderly population at the two study times and are unable to infer the causation of these trends. Thus, we propose to conduct prospective studies to examine the causes of the trends of hypertension prevalence, awareness, treatment and control among older persons in the future. Second, in the two surveys in this analysis, the diagnosis of hypertension is based on the BP measurement at a single visit on the examination day which might overestimate or underestimate the true prevalence and control of hypertension [37, 38]. Thirdly, the results in this study may be biased since those who more likely to respond to the survey may be more prone to being proactive about their health as compared to their counterparts. Despite these limitations, this study is the first population based study in Malaysia to track the trends of hypertension over 10 years period in two national surveys. The sample sizes of each study are large enough to establish trends of hypertension prevalence, awareness, treatment and control in the elderly population in Malaysia.

## Conclusions

The mean population BP and prevalence of hypertension among the elderly population in Malaysia have reduced significantly between 2006 and 2015. Although the awareness, treatment and control of hypertension among older persons have improved significantly, the awareness and control rates remain suboptimal. As population aging is inevitable, appropriate public health programs and optimal treatment strategies are urgently needed targeting this high risk group to improve the overall awareness and control of hypertension, with the ultimate goal of preventing the hypertension-related CVD complications.
